# Patient-engaged preoperative optimization for a complex incisional hernia: a case report

**DOI:** 10.3389/fsurg.2026.1871565

**Published:** 2026-09-09

**Authors:** Styliani Laskou, Theodora Gkeka, Panayiota Roulia, Athanasios Astreinidis, Leondias Kougias, George Geropoulos, Kyriakos Psarras, Konstantinos Sapalidis

**Affiliations:** 1 3rd Surgical Department, AHEPA University Hospital, Aristotle University of Thessaloniki, Thessaloniki, Greece; 2 Department of Radiology, AHEPA General University Hospital, Aristotle University of Thessaloniki, Thessaloniki, Greece

**Keywords:** abdominal wall reconstruction, botulinum toxin A, complex hernia, mesh, peritoneal flap hernioplasty, progressive pneumoperitoneum

## Abstract

Complex hernias with loss of domain present significant surgical challenges. Preoperative strategies such as botulinum toxin A (BTA) injections and progressive preoperative pneumoperitoneum (PPP) combined with structured prehabilitation and active patient participation may facilitate abdominal wall reconstruction. We report the case of a 65-year-old female with a complex incisional hernia. The patient underwent preoperative BTA injections followed by PPP before elective repair. This case highlights the value of a multimodal, patient-centered optimization pathway in facilitating successful abdominal wall reconstruction.

## Introduction

AWR has gained popularity over the last few years and is increasingly recognized as a surgical subspecialty. Despite the emerging interest in abdominal wall surgery and the employment of a more targeted-to-patient approach, the number of patients developing a hernia and undergoing repair is growing. Complex cases requiring AWR are also increasing. AWR represents a comprehensive reconstructive strategy aiming to restore abdominal wall anatomy and function. Although fascial release techniques are frequently required in complex cases, they are not mandatory in all reconstructions. Complexity is also attributable to the patients’ increased rates of co-morbidities and risk factors, which predispose them to a detrimental circle of complications and, hence, hernia recurrence (1, 2).

Consequently, it is strongly encouraged to optimize patients and deliver a technically proficient repair during the initial procedure. Preoperative prehabilitation along with adjuncts, such as botulinum toxin injection and progressive preoperative pneumoperitoneum (PPP), have been demonstrated to facilitate fascial closure in patients with or without loss of domain.

Herein we describe the management of a complex incisional hernia through a multimodal strategy engaging the application of preoperative (BTA), PPP, and structured prehabilitation, underlining the added value of patient engagement—an aspect increasingly recognized in the literature but often challenging to implement consistently in clinical practice.

## Case presentation

**Figure 1:**
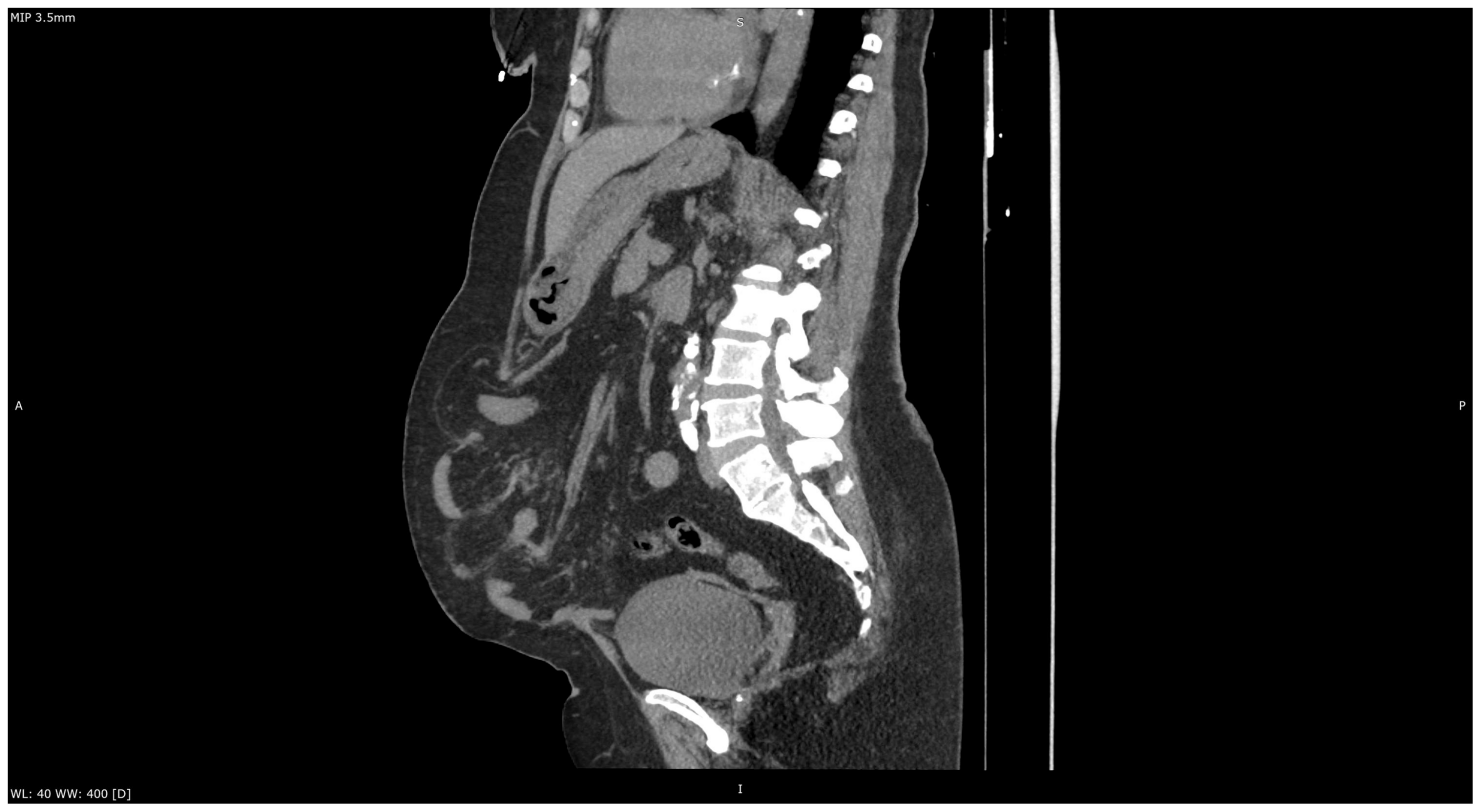
Preoperative sagittal CT scan demonstrating the large midline incisional hernia with herniation of small bowel and omentum through the abdominal wall defect. The image illustrates the hernia sac and reduced abdominal cavity volume before preoperative optimization.

A 65-year-old obese female (BMI: 33.1 kg/m^2^) presented to the outpatient department with abdominal bulging due to a large incisional hernia after open total hysterectomy with midline incision, 3 years ago. She had previously been evaluated at another institution before presenting to our department. Her medical history included arterial hypertension under treatment. She reported long-term tobacco use, estimated at about half a pack per day and essentially no routine physical activity. On clinical examination, a multi-orifice hernia was detected, not fully reducible but without any clinical signs of strangulation. Abdominal computed tomography revealed a 17 cm (length) × 10 cm (width), involving zones M2, M3, and M4 according to the European Hernia Society (EHS) classification (Figure 1). The loss-of-domain ratio, retrospectively calculated according to Tanaka's method, was approximately 25%, placing the patient at the lower threshold for consideration of progressive preoperative pneumoperitoneum.

After detailed counseling regarding the preoperative optimization strategy and the planned procedure, she agreed to postpone the procedure and actively engaged in prehabilitation. The prehabilitation program included supervised lifestyle optimization with emphasis on smoking cessation, nutritional counseling, and physical activity. The patient attended the institutional smoking cessation clinic and was referred to a dietitian to establish healthier eating habits with the goal of gradual preoperative weight reduction. She was encouraged to walk for at least 30 min daily. No pharmacological weight-loss therapy, including GLP-1 receptor agonists, was prescribed. Psychological support was available throughout both the outpatient optimization period and the subsequent inpatient admission to facilitate adherence to the treatment plan and perioperative preparation.

**Figure 2:**
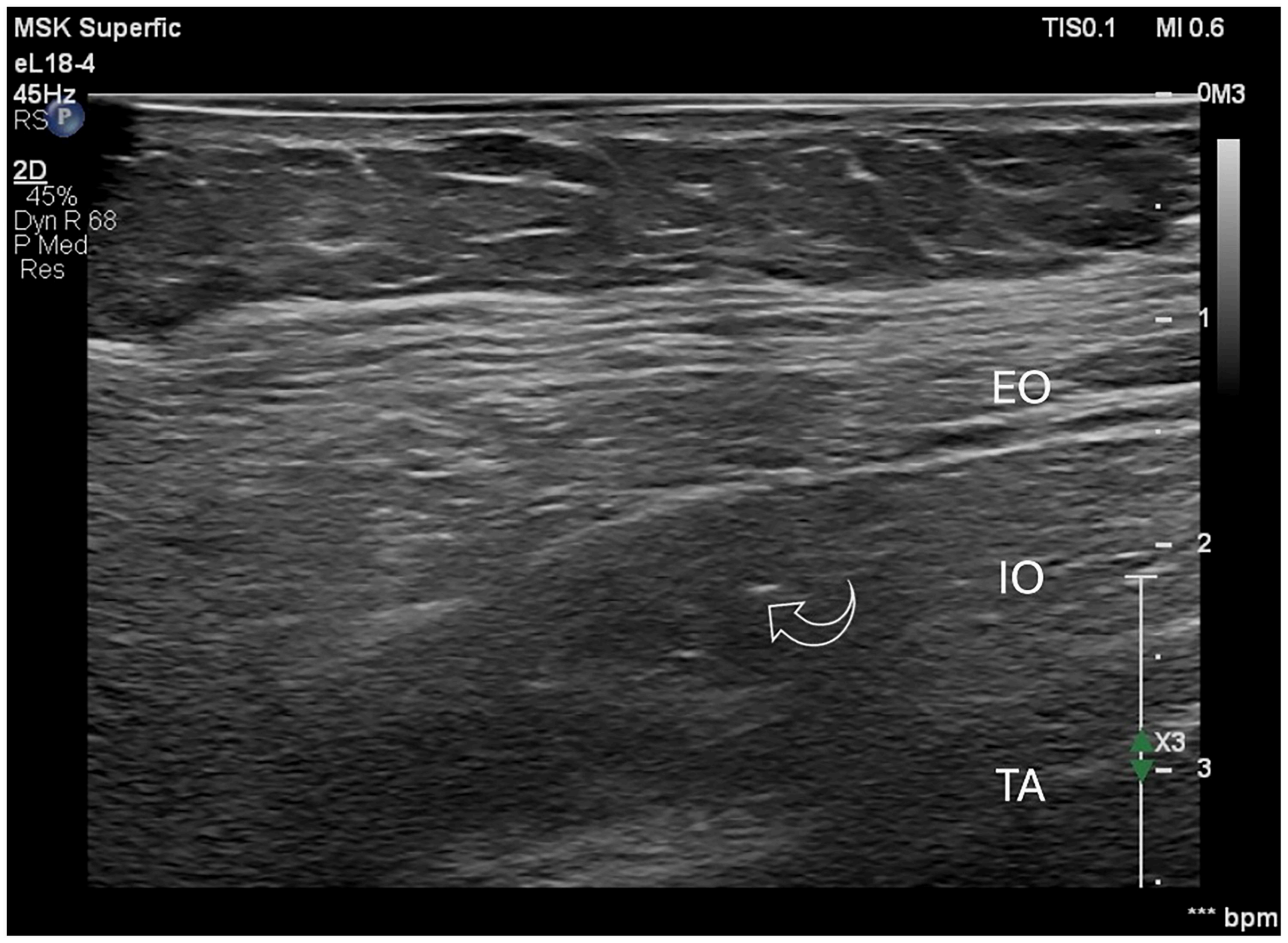
Ultrasound-guided botulinum toxin A injection. Ultrasound image demonstrating the injection needle (arrow) positioned within the transversus abdominis (TA) muscle, indicates the needle tip, and the solution is faintly visible at the tip. The three lateral abdominal wall muscle layers are identified: external oblique (EO), internal oblique (IO), and transversus abdominis (TA).

She was scheduled for BTA injection and PPP catheter placement. At our institution, BTA is considered in patients with large abdominal wall defects, retracted lateral musculature, or anticipated difficulty in achieving tension-free fascial closure, particularly when a more extensive component separation may otherwise be required. Six weeks prior to surgery, 300 units of BTA (Botox®) were injected under ultrasound guidance into three sites of each of the three lateral abdominal wall muscles (external oblique, internal oblique, and transversus abdominis) bilaterally to achieve preoperative muscle relaxation (Figure 2). As no complications were reported she was discharged the same evening.

Ten days preoperatively, the patient was readmitted and a CT was performed to acquire abdominal wall dimensions. Following BTA, the length of the lateral abdominal wall musculature increased from 140 (right) and 150 mm (left) to 170 and 165 mm respectively, while thickness decreased from 17 (right) and 23 mm (left) to 12 and 16 mm respectively.

Progressive preoperative pneumoperitoneum was initiated under CT guidance. Following percutaneous access to the peritoneal cavity with a 22-gauge spinal needle, 250 mL of room air was carefully insufflated and a confirmatory CT scan was obtained. Subsequently, an 8-Fr peritoneal catheter was positioned within the largest intraperitoneal air pocket identified on imaging. The patient remained hospitalized throughout the PPP protocol. Air insufflation was performed daily, with volumes adjusted according to individual tolerance and averaging approximately 1000 mL per day. Sessions were to be discontinued when abdominal discomfort, excessive distension, or respiratory symptoms such as dyspnea occurred. Pneumoperitoneum was maintained until the day of surgery.

**Figure 3:**
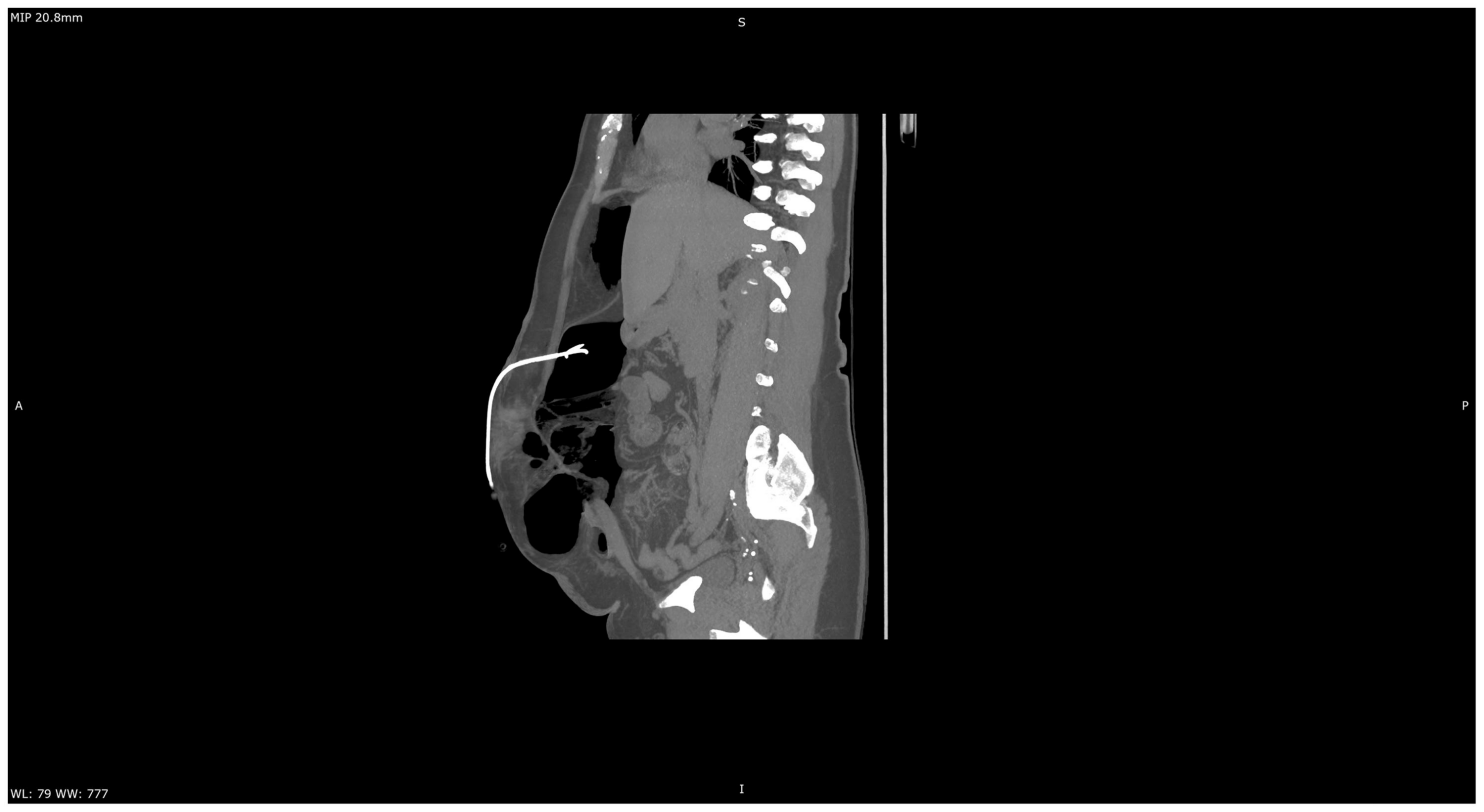
CT-guided progressive preoperative pneumoperitoneum (PPP). Sagittal CT image demonstrating the intraperitoneal catheter following successful placement and expansion of the abdominal cavity with ambient air during the PPP protocol.

During the treatment period, the patient received daily thromboprophylaxis with enoxaparin, which was discontinued 24 h before the operation. Additional preventive measures included the use of compression stockings and encouragement of regular ambulation (Figure 3).

**Figure 4:**
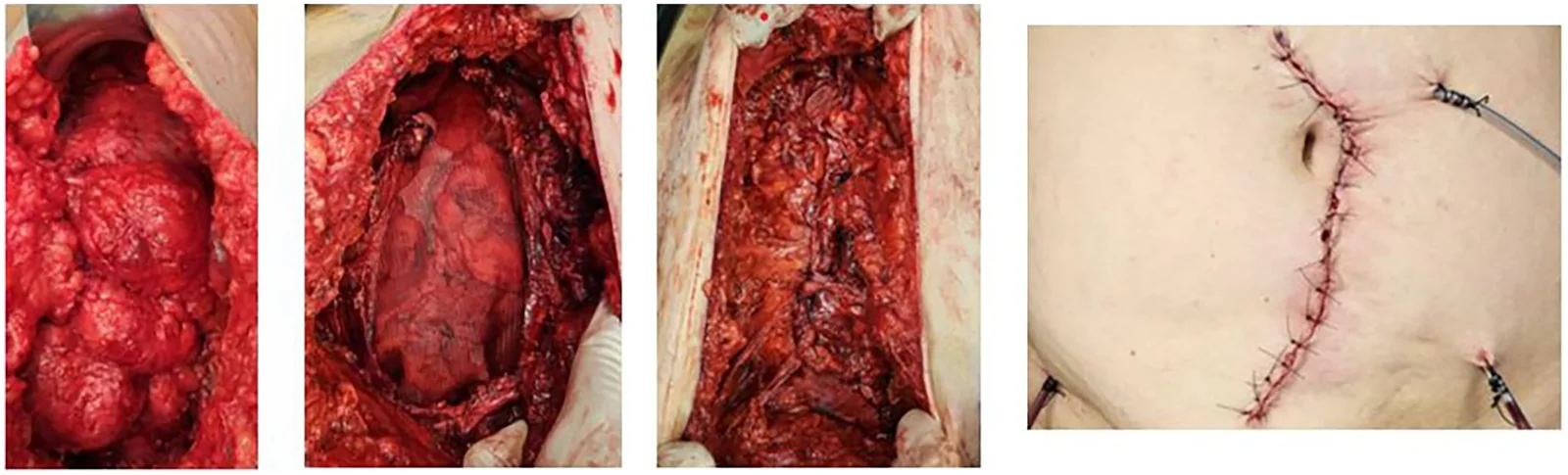
Intraoperative steps of abdominal wall reconstruction. Exposure of the large incisional hernia following elevation of the skin flaps. Reconstruction of the posterior layer using the preserved hernia sac before retromuscular mesh placement. Completed retromuscular reconstruction after mesh placement and anterior fascial closure. Final skin closure following panniculectomy.

The patient underwent open abdominal wall reconstruction through a midline laparotomy, including excision of the previous scar. The hernia sac was preserved throughout the dissection. Following development of the retromuscular plane from the xiphoid process superiorly to the Cooper's ligaments inferiorly, a portion of the sac was utilized to reconstruct the posterior layer, which was closed using a continuous 2-0 Vicryl suture. A 25 × 20 cm P4HB mesh was then placed in the retrorectus position. The anterior fascial layer was subsequently approximated in the midline without bridging using a continuous small-bites technique with 0 Vicryl. Panniculectomy was then performed to improve abdominal wall contour and minimize the risk of postoperative wound complications. Three closed-suction Redon drains were placed: two in the retromuscular space and 1 subcutaneously. They were removed once the drainage output was less than 30 mL over a 24-hour period (Figure 4). The total operative time was approximately three hours. Prophylactic incisional negative-pressure wound therapy was not available at our institution and therefore was not used.

**Figure 5:**
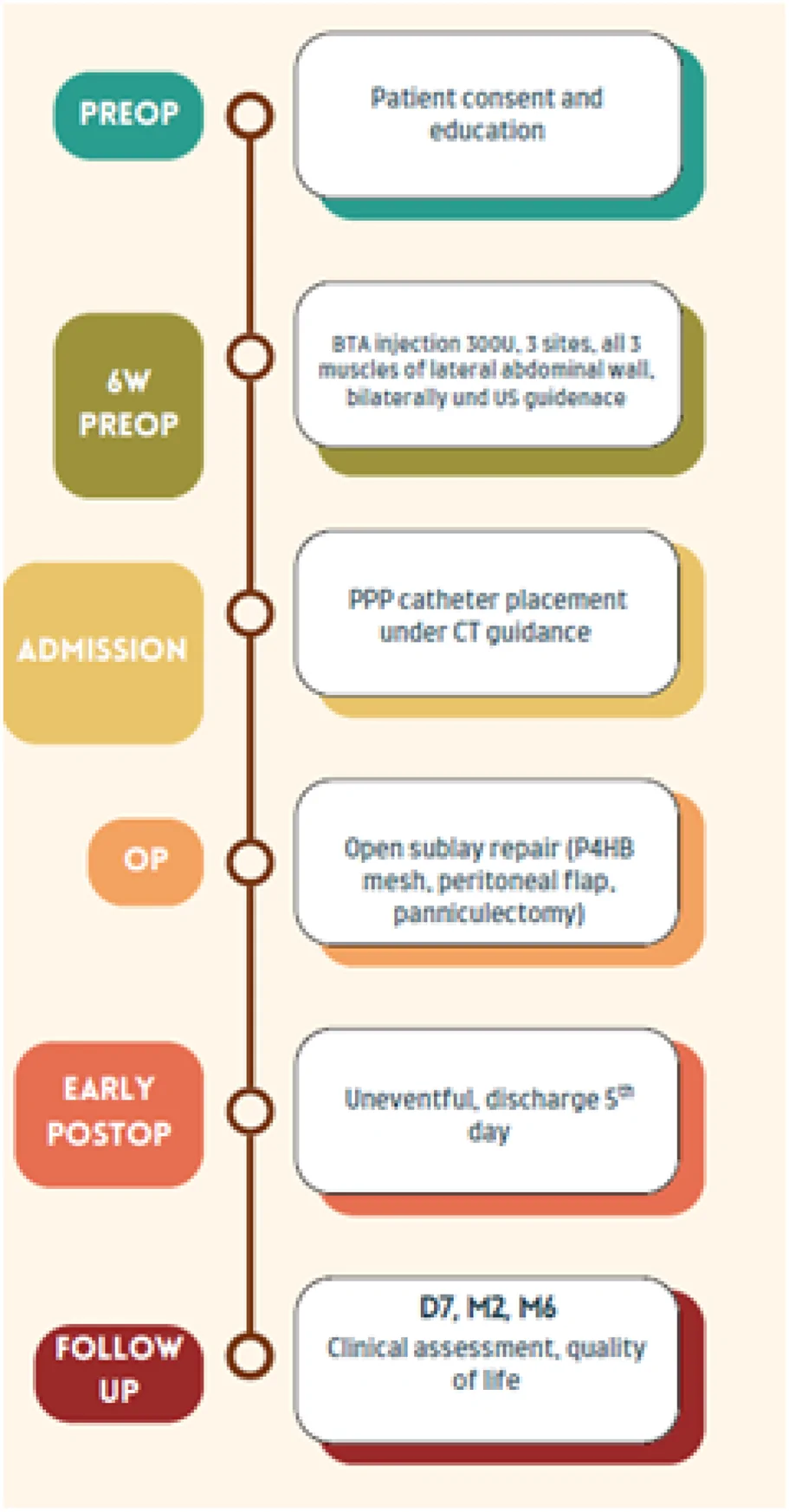
CARE timeline.

The postoperative course was uneventful, and the patient was discharged on the fifth postoperative day. Follow-up visits on postoperative days 7 and 15, and subsequently at 1, 2, and 6 months. At the first postoperative month, a seroma was observed, managed conservatively without the need for medical or surgical intervention (Clavien–Dindo Grade I). On re-evaluation satisfactory recovery without further complications or clinical evidence of recurrence was confirmed. Importantly, the patient reported a marked improvement in quality of life, with enhanced mobility and ability to perform daily activities, and her active participation in both prehabilitation and postoperative care was considered a key factor contributing to the favorable outcome. The complete chronological sequence of clinical events, interventions, and outcomes is summarized in Figure 5.

## Discussion

Managing incisional hernias is a well-recognized surgical challenge, often requiring a balance between technical feasibility and patient-related factors. The challenge becomes even greater when having to deal with complex incisional hernias. Such hernias are usually associated with large defects, multi recurrency and loss of domain which results from retracted lateral musculature and limited abdominal cavity volume. To facilitate tension-free abdominal wall reconstruction, various preoperative strategies have been proposed.

In the present case, a multimodal preoperative approach was applied. BTA when injected into the lateral abdominal wall muscles causes temporary flaccid paralysis serving as chemical component separation that allows gradual muscle elongation and facilitates fascial closure (3, 4). Timmer et al. provided a comprehensive review of the published injection techniques and treatment regimens summarizing that regardless of the trademark, BTA injections at 3–5 locations bilaterally in all three muscles of the lateral abdominal wall, about 4–6 weeks preoperatively is a safe procedure. Currently, this indication is off label (2, 4).

Synergistically with BTA, PPP enabled expansion of the abdominal cavity, promoted respiratory adaptation, and facilitated tension-free fascial closure while potentially reducing the risk of abdominal compartment syndrome (5). Pneumoperitoneum catheters may be inserted laparoscopically or under CT guidance, as in the present case (6). Several PPP protocols have been described in the literature, differing in catheter placement, insufflation volume, treatment duration, and inpatient versus ambulatory management (6, 7). Recent studies have demonstrated that ambulatory PPP is feasible in carefully selected patients (7). However, in the present case, PPP was performed on an inpatient basis because the patient resided a considerable distance from our institution, allowing daily clinical assessment, individualized adjustment of insufflation volumes according to tolerance, and close monitoring for potential complications. Although the calculated Tanaka index was approximately 25%, representing a borderline indication for PPP, we elected to combine PPP with BTA in an effort to downstage the reconstruction, facilitate tension-free closure, and potentially avoid more extensive component separation techniques such as TAR. Our intention was not to avoid TAR *per se*, but rather to maximize the effectiveness of preoperative optimization strategies and reserve more extensive component separation techniques for situations in which satisfactory fascial closure could not otherwise be achieved. During hospitalization, prophylactic enoxaparin was administered according to our institutional protocol because of the patient's obesity, anticipated reduced mobility, and prolonged inpatient stay. Close monitoring for complications such as subcutaneous emphysema, pneumothorax, or catheter-related problems was maintained throughout treatment (1).

Both interventions require careful planning and patient compliance. While prehabilitation and patient engagement have been increasingly studied, uncertainty remains regarding the magnitude of their effect on postoperative outcomes and successful implementation in everyday clinical practice. Prehabilitating patients seems to decrease the risk for wound and pulmonary complications (8, 9). Fafaj et al, however, wonders whether these measures truly enhance surgical outcomes or simply postpone the inherent difficulties of complex abdominal wall reconstruction as well as let patients suffer (10). Prehabilitation programs should aim to increase patients’ physiological reserve by a combination of exercise, smoking cessation, nutritional optimization. Evidence from systematic reviews and meta-analyses propose that such interventions can improve functional capacity and reduce postoperative pulmonary and overall morbidity in major abdominal surgery (11–13). Published evidence for abdominal wall surgery is more limited but the existing papers highlight the potential role in reducing complications and improving recovery (14, 15). As far as the optimal duration is concerned, most protocols report a 2-to-6-week period while approximately 4 weeks are often considered sufficient to achieve measurable improvements in functional capacity (16–19).

In the present case, the combination of active participation and willingness to undergo a period of preparation ultimately translated into a successful repair, a smooth postoperative course and a meaningful improvement in quality of life. Beyond the benefits of prehabilitation, the reconstruction was further facilitated using a sublay P4HB mesh. Such a mesh, according to a multicenter clinical trial, achieves acceptable recurrence rates detected by radiological follow-up. During a 5-year period, a recurrence rate of 15.9% in high-risk patients was observed (20). Other important outcome parameters, including mesh-related complications and chronic pain, also appear to be favorably influenced, with lower rates of long-term inflammatory response and improved patient comfort compared to conventional synthetic materials (20, 21). In the present patient, obesity, previous smoking history, extensive abdominal wall dissection, and concomitant panniculectomy were additional factors supporting the choice of a biosynthetic mesh. Furthermore, the favorable remodeling profile and reduced foreign-body sensation reported in the literature were considered potential advantages. Nevertheless, authors, although emphasizing the feasibility and safety of P4HB mesh, declare that longer follow-up periods and studies with larger populations are needed to more definitively assess long-term outcomes.

In conclusion, the present case was not intended to demonstrate the novelty of individual techniques such as botulinum toxin A administration or progressive preoperative pneumoperitoneum, both of which are well established in the management of complex abdominal wall defects. Rather, its educational value lies in illustrating how a multimodal, patient-centered optimization pathway—including structured prehabilitation, smoking cessation, nutritional counseling, psychological support, patient engagement, BTA, PPP, and tailored surgical reconstruction—can be integrated into a single treatment strategy. We believe that careful patient selection and comprehensive preoperative optimization may facilitate tension-free fascial closure while potentially reducing the need for more extensive myofascial release procedures. Larger prospective studies are warranted to determine the reproducibility and broader clinical impact of this approach.

## Data Availability

The original contributions presented in the study are included in the article/Supplementary Material, further inquiries can be directed to the corresponding author.
